# [Retracted] MicroRNA‑574‑3p inhibits the malignant behavior of liver cancer cells by targeting ADAM28

**DOI:** 10.3892/ol.2023.13973

**Published:** 2023-07-24

**Authors:** Zhongming Zha, Fuxin Jia, Pingan Hu, Erhui Mai, Ting Lei

Oncol Lett 20: 3015–3023, 2020; DOI: 10.3892/ol.2020.11852

Following the publication of this article, a concerned reader drew to the attention of the Editorial Office that the flow cytometric images shown in Fig. 2E were strikingly similar to flow cytometric data that had already been submitted for publication in different form in Fig. 2D of the following paper, written by different authors (albeit one research institute was held in common between the two papers): Chang K, Wei Z and Cao H: miR-375-3p inhibits the progression of laryngeal squamous cell carcinoma by targeting hepatocyte nuclear factor-1β. Oncol Lett 20: 80, 2020].

Owing to the fact that the contentious data in Fig. 2 of the above article were already under consideration for publication prior to its submission to *Oncology Letters*, the Editor has decided that this paper should be retracted from the Journal. After having been in contact with the authors, they accepted the decision to retract the paper. The Editor apologizes to the readership for any inconvenience caused.

